# The PULSAR primary care protocol: a stepped-wedge cluster randomized controlled trial to test a training intervention for general practitioners in recovery-oriented practice to optimize personal recovery in adult patients

**DOI:** 10.1186/s12888-016-1153-6

**Published:** 2016-12-20

**Authors:** Joanne C. Enticott, Frances Shawyer, Lisa Brophy, Grant Russell, Ellie Fossey, Brett Inder, Danielle Mazza, Shiva Vasi, Penelope June Weller, Elisabeth Wilson-Evered, Vrinda Edan, Graham Meadows

**Affiliations:** 1 Southern Synergy, Department of Psychiatry, Monash University, 126 Cleeland St, Dandenong, VIC Australia; 2 Royal District Nursing Service Institute, 31 Alma Rd, St Kilda, VIC Australia; 3 Mind Australia, Heidelberg, VIC Australia; 4 Melbourne School of Population and Global Health, University of Melbourne, Parkville, VIC Australia; 5 School of Primary Health Care, Monash University, Notting Hill Campus, Victoria, Australia; 6 Southern Academic Primary Care Research Unit, Monash University, Notting Hill, Victoria, Australia; 7 School of Primary Health Care, Monash University Peninsula Campus, Frankston, VIC Australia; 8 Department of Econometrics and Business Statistics, Monash University, Melbourne, VIC Australia; 9 Graduate School of Business and Law, RMIT University, Melbourne, VIC Australia; 10 College of Business, Victoria University, Melbourne, VIC Australia; 11 Monash Health, Melbourne, VIC Australia

**Keywords:** Recovery, Recovery-oriented Practice, Primary Care, General Practitioners, Mental Health, Psychiatry, Training, Randomized Controlled Trial (RCT), Complex Intervention

## Abstract

**Background:**

General practitioners (GPs) in Australia play a central role in the delivery of mental health care. This article describes the PULSAR (Principles Unite Local Services Assisting Recovery) Primary Care protocol, a novel mixed methods evaluation of a training intervention for GPs in recovery-oriented practice. The aim of the intervention is to optimize personal recovery in patients consulting study GPs for mental health issues.

**Methods:**

The intervention mixed methods design involves a stepped-wedge cluster randomized controlled trial testing the outcomes of training in recovery-oriented practice, together with an embedded qualitative study to identify the contextual enablers and challenges to implementing recovery-oriented practice. The project is conducted in Victoria, Australia between 2013 and 2017. Eighteen general practices and community health centers are randomly allocated to one of two steps (nine months apart) to start an intervention comprising GP training in the delivery of recovery-oriented practice. Data collection consists of cross-sectional surveys collected from patients of participating GPs at baseline, and again at the end of Steps 1 and 2. The primary outcome is improvement in personal recovery using responses to the Questionnaire about the Process of Recovery. Secondary outcomes are improvements in patient-rated measures of personal recovery and wellbeing, and of the recovery-oriented practice they have received, using the INSPIRE questionnaire, the Warwick-Edinburgh Mental Well-being Scale, and the Kessler Psychological Distress Scale. Participant data will be analyzed in the group that the cluster was assigned to at each study time point. Another per-protocol dataset will contain all data time-stamped according to the date of intervention received at each cluster site. Qualitative interviews with GPs and patients at three and nine months post-training will investigate experiences and challenges related to implementing recovery-oriented practice in primary care.

**Discussion:**

Recovery-oriented practice is gaining increasing prominence in mental health service delivery and the outcomes of such an approach within the primary care sector for the first time will be evaluated in this project. If findings are positive, the intervention has the potential to extend recovery-oriented practice to GPs throughout the community.

**Trial registration:**

Australian and New Zealand Clinical Trial Registry (ACTRN12614001312639). Registered: 8 August 2014.

## Background

### Australian primary care and mental health

Most Australian medical general practitioners (GPs) work within privately owned general practices where they play an important direct role in the diagnosis and management of mental health problems [1]; around 13% of GP consultations are related to mental health, with depression cited as the most common illness [2, 3]. Use of GP services directly or through prescribing contributes substantially to the over $8 billion per annum cost of mental health services [4], and Australian GPs also have specific roles in determining eligibility to many of the specialist services that make up the rest of this cost [5]. Referrals for mental health, as a percent of total GP encounters, are growing through time with an annual average increase of 7% in the five years up to 2013/14 [6].

In the State of Victoria, Australia, the arrangement for mental health services reflects the hybrid national system of mixed private and public service delivery. Australian Medicare, intended as a universal health insurance scheme, provides Australian residents the right to assign specified benefits to private health providers, including GPs, based on items of care including consultations. Co-payments are unrestricted and practitioners can choose their location of practice. The majority of people in Victoria with mental health issues access mental health services via their GP [7]. State-run public sector specialist clinical mental health services, typically accessed by people with more severe mental illnesses, are block-funded. Of all third-party expenditure on mental health care delivery, 61.0% is through State and Territory Governments, 35.2% from the Commonwealth Government, the remainder from private insurance [8]. In Victoria, state-funded mental health care includes a substantial investment in the non-clinical community mental health support sector (run by non-government organizations) which provide programs that help individuals manage their own recovery and maximize their involvement in community life [7].

### Recovery-oriented practice, REFOCUS and the PULSAR project

An approach to mental health care that has gained momentum over recent years is recovery-oriented practice, which involves facilitating a process of change through which individuals are supported to build and live fulfilling and meaningful lives, with or without the continuing presence of mental health issues [9, 10]. The meaning of the term ‘recovery’ in this context is distinguished from clinical recovery and has been summarized as:

*A deeply personal, unique process of changing one’s attitudes, values, feelings, goals, skills and roles. It is a way of living a satisfying, hopeful and contributing life even with limitations caused by the illness. Recovery involves the development of new meaning and purpose in one’s life as one grows beyond the catastrophic effects of mental illness* [11].

A paradigm shift towards recovery-oriented practice in specialist mental health service delivery is being embraced internationally [12–14]. An evidence-based package of tools, developed [15] and trialed [16] by the REFOCUS Team (Institute of Psychiatry, King’s College London) and known as the REFOCUS Intervention, has been used to promote recovery-oriented practice in specialist mental health teams in the UK. In Australia, recovery-oriented practice has been endorsed through the Australian National Mental Health strategy from the early 2000s [17] with various efforts to promote a recovery framework made in the eight States and Territories [18]. While there is extensive work on recovery-oriented practice in specialist service delivery, a focus on personal recovery is less well established as influential in GP training and practice. Moreover, to our knowledge, no trial has yet been published in Australia or elsewhere that has examined whether interventions promoting recovery-oriented practice in primary care improve outcomes for patients. Given that the GP is the service provider most commonly consulted for mental health problems [19], this represents a critical gap.

The Principles Unite Local Services Assisting Recovery (PULSAR) Primary Care project is part of the broader PULSAR research program into approaches to promoting recovery-oriented practice. PULSAR involves adaptation of REFOCUS materials [15, 20] for Australian primary and specialist care [21]. For the PULSAR Primary Care project, the REFOCUS materials were adapted for Australian privately owned GP practices and government funded community health centers. Introduction of these principles and practices into primary care with associated research is part of the scope of the PULSAR project and described in this paper. It might be noted that although formal introduction of recovery-oriented practice to general practice may be innovative, some elements may not be entirely novel to GPs since recovery-oriented practice can be seen as having common ground with influential concepts in primary care including, for instance, Shared Decision Making [22–24], Patient Centered Care [25], and a doctor–patient relationship characterized by Mutual Participation [24]. We also note at this point that in description of parallel PULSAR Secondary Care projects we refer to people who experience mental illness and engage with services as ‘consumers’ but in the Primary Care project described in this paper, the term ‘patient’ will be used as more representative of regular usage in this context.

This paper describes the PULSAR Primary Care study protocol. The protocol adheres to the SPIRIT (Standard Protocol Items: Recommendations for Interventional Trials) 2013 guidelines [26].

### Aims and objectives

The aim of the PULSAR Primary Care study is to conduct a mixed methods evaluation of a training intervention for GPs in recovery-oriented practice. The purpose of the intervention is to optimize personal recovery in patients consulting project study GPs for mental health issues. The project employs an intervention mixed methods design [27], involving a stepped-wedge cluster randomized controlled trial (cRCT) of training in recovery-oriented practice, together with an embedded qualitative study of implementing recovery-oriented practice in primary care. Clusters are participating general practices and community health centers that employ GPs. The objective of the quantitative research is to examine whether adult patients of GPs and practices that have received training in recovery-oriented practice report greater personal recovery compared to other patients where practices have not received the intervention. The following research questions are addressed:

1. From pre- to post-intervention, do patients of intervention cluster GPs report greater improvements on measures of personal recovery compared with control group patient participants?
2. From pre- to post-intervention, do patients of intervention cluster GPs report greater improvements on measures of health and wellbeing status compared with control group patient participants?
3. From pre- to post-intervention, do patients of intervention cluster GPs report greater improvements on measures of perceived need for care and satisfaction with services compared with control group participants?
4. Are any changes in estimated service costs compatible with favorable health economic properties for the intervention?
5. What are the contextual enablers and challenges to implementing recovery-oriented practice in primary care settings?

## Methods

### Overall design

**Fig. 1:**
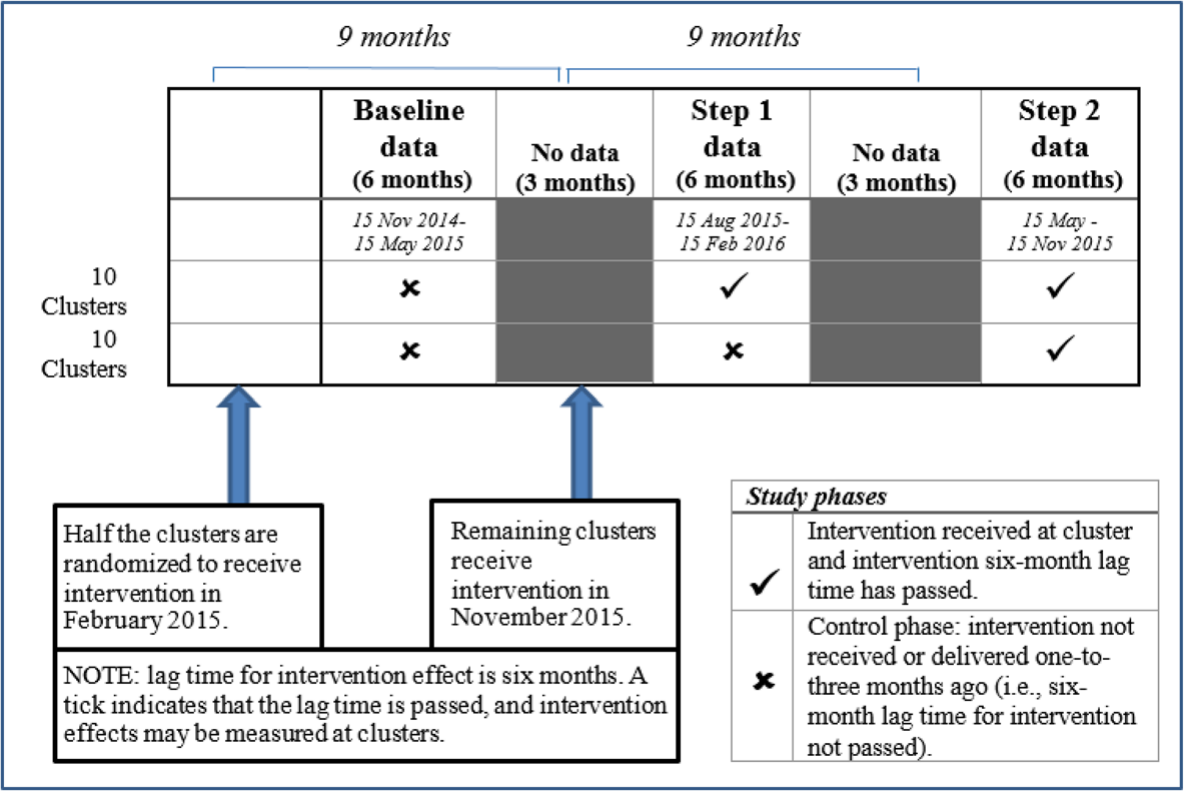
A stepped-wedge cluster randomized controlled trial in general practice and community health center primary care settings. Data collection from patients is planned to occur during the six-month periods at baseline, Step 1, and Step 2

The intervention mixed methods design involves a stepped-wedge cluster randomized controlled trial testing the outcomes of training in recovery-oriented practice, together with an embedded qualitative study to identify the contextual enablers and challenges to implementing recovery-oriented practice within primary care settings. Eighteen general practices and community health centers were randomly allocated to one of two steps (9 months apart) to start an intervention comprising general practitioner training in the delivery of recovery-oriented practice. Data collection consists of cross-sectional surveys collected from patients of participating general practitioners at three time points: baseline, and again at the end of Steps 1 and 2 (see Fig. 1). A mixed methods design was chosen for a number of reasons, including that it would produce richer and more complete findings than that based on either the qualitative or quantitative approach alone. Combining these approaches can further explain and enhance the integrity of the project outcomes, so that a mixed methods design may assist with project credibility, particularly when multiple stakeholders are involved such as in the PULSAR project [28]. The use of qualitative and quantitative methods were integrated at different stages of the intervention trial [27]. Initially, qualitative methods were used in an exploratory manner to identify potential barriers to recovery-oriented training within primary care settings, and to inform the intervention design. Subsequently, quantitative methods are being used to then measure the effectiveness of the intervention. Following the intervention trial, qualitative methods will be used in an explanatory manner to gain a more nuanced understanding of GP experiences of implementing recovery-oriented practices and patient experiences of these practices [28]. Quantitative and qualitative data are collected from patient participants, but only qualitative data are collected from GP participants.

A process evaluation was nested within the study design to collect qualitative, quantitative, and documentary data. The purpose of the process evaluation is to explain those factors that impinge on the study design and how processes and decisions during the course of the study could explain or elaborate on findings. Process evaluation is often missing from randomized controlled trials; however, the UK Medical Research Council has provided useful guidelines for bringing rigor to the conduct of such evaluations. The value of process evaluation within trials such as the PULSAR study is to assess fidelity and quality of implementation, clarify causal mechanisms and identify contextual factors associated with variation in outcomes [29] These guidelines inform the approach in the current study [29] which includes analysis of process of implementing a complex intervention design associated with the stepped-wedge approach, sampling responses, effect sizes, and organizational and contextual factors that impact the study processes and outcomes. The process evaluation draws on the findings of the UK REFOCUS process evaluation [30] which explored service users’ experience of receiving a complex pro-recovery intervention. Furthermore, Bhanbhro and colleagues’ [31] recent work confirmed the crucial importance of taking account of practitioner, trainer and organizational factors when implementing a training intervention to achieve practice change in diverse mental health settings [31].

The process evaluation will focus on the following:

1. Service users’ experience of the PULSAR training and PALS intervention
2. The implementation context
3. The research process and engagement
4. The impact and outcomes of the study.

### Explanation for choice of comparators

The design was developed to combine the rigor of a cluster randomized trial with the pragmatic approach of the stepped wedge design to implement the intervention at all sites [32–34]. Control sites are those that are yet to receive the intervention. Since all sites eventually receive the intervention, data from sites in control phases is to be compared with data from sites that have received the intervention. There are no study restrictions on the care provided in control phases.

### Study setting

Recruitment of cluster sites and GPs was initially to be within the Monash Health catchment in Victoria, Australia [35]. However, challenges in recruiting GPs emerged when this geographical restriction was applied, so recruitment was extended to include any cluster site in the greater Monash Health region and adjacent areas. This encompasses the catchment areas of three Medicare Locals that overlap with the Monash Health catchment. Medicare Locals were organizations established to coordinate the delivery of primary health care within bounded geographical regions so as to address local health care priorities and improve access to primary care. These were decommissioned during the project (30 June 2015) and replaced with Primary Health Networks, which created challenges for ongoing engagement with GPs.

The study setting was expanded to include the following local government areas: City of Monash, City of Greater Dandenong, City of Casey, Cardinia Shire, City of Kingston, City of Glen Eira, City of Bayside, City of Frankston, Knox City, and the Shire of Mornington Peninsula. The final study setting area consists of approximately 1.392 million Victorians, or 24% of Victoria’s population [35]. It includes affluent areas and semi-rural growth corridors as well as the most socially and financially disadvantaged area in metropolitan Melbourne (the City of Greater Dandenong), with 56% of its residents born overseas and high numbers of refugees [36]. It also includes areas with disproportionately high numbers of retirees and older Australians in the Shire of Mornington Peninsula.

A list of the final participating study sites can be obtained from the contact author, but only if sites consent to this.

### Eligibility criteria

#### GP criteria

To be eligible, practice sites are required to meet the accreditation standards for quality of care and risk management set by the Royal Australian College of General Practitioners [37]. GP eligibility criteria are: having worked at their current practice for at least 12 months, with minimum 2.5 days per week at the study site and a majority of that work in generalist primary care. Enrolment involves committing to participate in the recovery-oriented training intervention, to identify eligible patients and to distribute study invitation letters and surveys to potential patient participants.

#### Patient criteria

Base inclusion criteria for patient participants are:

- aged 18 years and over
- aged less than 75 years of age
- proficient in English
- able to provide informed consent
- patients of a participating GP, that is, consult with the GP in 50% of their visits to the practice or is identified by the GP as a patient.

In addition to the base criteria, of the ten patients to be recruited at each of the 3 time periods (see Fig. 1), seven are to have at least one of the following:

- a recent mental health plan made, or
- a review of a mental health plan, or
- prescribed any class of antidepressant medication on a continuing basis as treatment for a mental illness; and

three are to have at least one of the following:

- a diagnosis of psychosis (e.g., schizophrenia, schizoaffective disorder, bipolar disorder), or
- been prescribed antipsychotic medication in the previous 6 months

Individuals in prison are excluded.

### Intervention

The intervention consisted of training modules and optional ongoing participation in monthly webinars with participating GPs. Before development of the training materials, individual semi-structured interviews with GPs explored potential barriers to recovery-oriented training within primary care settings and this information was used to assist in design. The training intervention was designed in alignment with the modular training pathway of the General Practice Mental Health Standards Collaboration Mental Health Skills Training (MHST) requirements [38] and received accreditation for this as well as accreditation through the two relevant Australian GP Professional Colleges. Patients of GPs who have completed MHST can claim higher rebates for some mental health care activities Medicare.

The two modules of the recovery-oriented training intervention delivered to participating GPs are: 1. Core Module (3.5 h) with learning objectives including increased skill in recognizing and assessing common mental illnesses within a recovery-oriented framework; a greater working knowledge of the Better Access initiative and mental health treatment planning with a focus on operationalizing recovery-oriented practice in general practice; enhanced understanding of the perspective of consumers and carers in the provision of mental healthcare; and increased knowledge of the local mental healthcare services and resources available to GPs; and 2. Clinical Enhancement Module (CEM; 4 h), which provides the opportunity to apply knowledge gained in the Core Module within the specific context of Schizophrenia. Learning objectives of the CEM include: development of skills in the detection and assessment of Schizophrenia; an ability to apply the principles of recovery-oriented practice to treatment planning and monitoring; the ability to develop recovery-focused mental health treatment plans; and an applied understanding of review processes and relapse prevention strategies for mental illness within a recovery-oriented practice framework. MHST criteria are met by participation in both modules, along with preparatory and reinforcing exercises. For GPs who already have MHST accreditation, the option is open to attend the CEM only, and one round of the training is delivered as something of a hybrid between the two but with full retention of the recovery-oriented practice content. In the latter two situations, GPs retain access to professional development points through their college but the MHST requirements do not need to be met.

In addition to GPs, other clinic/practice staff such as practice nurses, non-participating GPs, and administration staff are also encouraged to attend training. Training is delivered by mental health clinicians, including experienced trainers from the study team, with co-delivery by consumer trainers. An experienced family/carer worker also participates, providing a minimum of one hour of content over the total of eight hours of training. The intervention draws heavily on the REFOCUS program [15] and the General Practitioner Mental Health Treatment Plan-Recovery (GPMHTP-R). The latter was developed in the Better Mental Health Treatment Plans Project, which was conducted by the Southern Synergy group with the support of a Commonwealth Government funding grant. The recovery-oriented training intervention includes training in the use of the GPMHTP-R, and also features locally developed materials. The intervention was developed following consultation and discussion with a group of local GPs (*n =* 7).

Each training participant is provided with resource material consisting of background reading and the Primary Care PULSAR Manual, which explains how to incorporate recovery-oriented practice into regular practice. The Project Team is responsible for developing, evaluating, and making these resources readily available. A schedule of training is provided to trainers, along with standardized training packs (including videos) to ensure the provision of standardized content. Trainers are asked to keep a record of training and to record any deviations from the schedule and use of materials.

### PALS: PULSAR active learning sessions

GPs and other professionals who have received the PULSAR training are invited to participate in monthly one-hour online sessions called “PALS (PULSAR Active Learning Sessions)” with consultant specialist psychiatrists to review, reflect and share their experiences in the implementation of recovery-oriented practice. These sessions provide an interactive learning environment for supporting practice-based implementation of learning from the PULSAR resources and training package.

#### Intervention modifications and delays

Any delays in implementing the training during the protocol timelines are noted and the actual date of training recorded. The analysis plan has been expanded to include all available data with the actual training date in an attempt to maximize the informational value from the collected data (see *Statistical analysis* section).

#### Dosage

To account for potential reduction of the intervention ‘dosage’ within each cluster, dosage is measured as the percentage of GPs who undergo the recovery-oriented practice training. This variable is based on the percentage of cluster staff who complete the training and remain in the cluster. For example, if two GPs at a cluster receive the training intervention at Step 1 and both remained employed at the practice/clinic/health center, this dosage variable would be 100% at Step 2.

#### Access to study intervention at study closure

At the end of the study period GPs and their staff will continue to have access to the training resources provided, except for PALS. It is anticipated that the resources developed will inform ongoing initiatives by the local Primary Health Networks.

### Randomization

Clusters were randomized to receive the intervention at either Step 1 or Step 2 (see Fig. 1) using a minimization procedure [39]. To ensure that each step period had a balance of cluster types, stratified randomization was applied using four types of organizational variations (see the cluster level stratification variables in Table 2). The study statistician performed the stratified randomization through December 2014 to June 2015, and sites were notified shortly afterwards.

### Breaking the cluster intervention code

Breaking the cluster intervention code necessarily occurred after randomization (see above) so that training could be organized at the clusters. However, only key people involved with the organization and delivery of the training were informed of the intervention/training schedule.

### Quantitative methods

#### Design

The original study protocol (as documented in the Australian New Zealand Clinical Trials Registry, or ANZCTR) was developed over a period of 18 months and involved consultation with the Chief Investigators (CIs) and local stakeholders, and included representation from primary care, community care, and patients. The planning team included primary care academics familiar with the known barriers associated with recruitment of GPs into research projects [40–42].

The research team originally planned to deliver the recovery-oriented practice training intervention to one participating GP per cluster site, and to evaluate the process using a study design of a cRCT conducted over four years, utilizing a two-step stepped-wedge design. This design was developed to combine the rigor of a cluster randomized trial with the pragmatic approach of the stepped-wedge design to implement the intervention at all sites. However, initial challenges with GP engagement and patient recruitment resulted in necessary modifications to the original project protocol. These are outlined below. All adaptations to the study protocol were considered by, and required the approval of, the appropriate Module Committee governing the relevant aspect of the project (see *Study leadership* section). Half-yearly project reports to the funding body also advised of changes made or intended. In each case these were then considered by the agent of the funding body. Queries as raised in some cases were responded to by the investigators prior to acceptance of the report by the funding body agents. Adaptations to the original protocol as reported here were thereby given endorsement from the funding body. Despite the adaptations described below, the basic two-step stepped-wedge cRCT design remains unchanged (see Fig. 1) and the trial will conclude within the planned four-year timeframe.

#### Adaptations to design

Delays in recruitment of GPs and patients and in delivery of the intervention required some modification to the design in order to be able to deliver the project outcomes within the allowed project duration. Cluster definition was broadened to include one or more GPs from each site in an effort to boost recruitment. As well, study steps were reduced from 12 months to 9 months. Based on the Kirkpatrick training evaluation model [43], the 9-month step period is an acceptable time point for the measurement of change. It allows 3 months for consolidation and a 6-month period for patients to be exposed to GPs implementing recovery-oriented practice.

#### Measures

All outcome measures are listed in Table 1, and the study survey is available upon request to the contact author. The primary outcome measure, the 22-item Questionnaire about the Process of Recovery (QPR) [44], enables an examination of the primary research question by collecting information from patients of participating GPs about personal recovery. This patient-rated outcome measure was chosen for the study end-point as personal recovery is experienced by an individual rather than assessed by an expert.

**Table 1:** Primary, secondary and other outcome measures in PULSAR Primary Care

Patient survey (quantitative) measures	
Primary outcome	1. Questionnaire about the Process of Recovery (QPR) [44]
Secondary outcomes	2. INSPIRE questionnaire [45] 3. Warwick-Edinburgh Mental Well-being Scale (WEMWBS) [46] 4. Kessler Psychological Distress Scale (K10) [47]
Other measures	5. Participant Demographic Record 6. General-practice-Users Perceived-need Inventory (GUPI) [48] 7. Health economic and service utilization questionnaire
Patient qualitative data	
Interviews	Individual interviews
Focus groups	Focus groups
GP qualitative data	
Interviews	Individual interviews
Focus groups	Focus groups

The secondary outcome measures include the importance of services in recovery questionnaire (INSPIRE) [45], which assesses the recovery orientation of the GP service from the perspective of the patient, and two measures of mental health and wellbeing, the Warwick-Edinburgh Mental Well-being Scale (WEMWBS) [46], and the Kessler Psychological Distress Scale (K10) [47].

Additional measures are: Participant Demographic Record and the General-practice-Users Perceived-need Inventory (GUPI) [48]. As well, a section in the survey is designed to gain an understanding of any financial burden of illness as well as the participants’ use of health services. These health economic and service utilization questions, which contain both study-specific items and questions adapted from the 2007 Australian National Survey of Mental Health and Wellbeing [49], include information on the participants’ job status, income, consultations with health professionals, medications being taken, admissions to hospital, and any time away from usual roles because of illness. Participants are advised that if they cannot answer a question precisely, then to provide a best estimate and that there are no ‘right’ or ‘wrong’ answers. Finally, where specific consent is provided, information related to diagnosis, mental health status and current medication is also collected from GP medical records.

#### Sample size targets

The study has two sample groups: GPs and patients of these GPs. Sample size calculations were done using Stata statistical software *stepped-wedge*: for clusters defined at the level of the general practice site, the primary outcome measure is the QPR, with a published mean of 46 and standard deviation of 16, power of 0.80, significance level set at 0.05, intraclass correlation coefficient (ICC) within practice sites of 0.05, the number of steps (2), and patient data collected cross-sectionally at three time points (baseline, Step 1, and Step 2).

The original sample target was a minimum of 20 different sites (clusters) with one GP per site; each GP was required to recruit a minimum of 30 patients during the four-year project (10 patients at baseline, 10 at Step 1, and 10 at Step 2), resulting in a minimum of 600 patient participants overall.

#### Adaptations to sample size targets

Challenges in engaging GPs and recruiting patients were encountered at the beginning of the project (see earlier and also below). As a result, the protocol was adapted so that more than one GP from each cluster can participate. Each participating GP is still expected to recruit a minimum of 30 patients over the duration of the trial; therefore, if there are two participating GPs at a cluster, each will recruit 30 patients, resulting in 60 patients for that cluster. This efficiency of scale appears to be working well in some clusters. The required cluster number was also reduced from 20 to 10 plus. These sample numbers will be sufficient to detect a medium to medium-large effect in the primary outcome.

#### GP recruitment

Over 325 general practice and community health centers in the Monash Health catchment area were identified by the research team, using the National Health Services Directory and the telephone directory. The team also accessed a comprehensive database of GPs who have been involved in GP research in the south-east region of Melbourne held by the Department of General Practice at Monash University. Existing contacts within relevant health care organizations that coordinate service delivery related to primary care, such as the Medicare Locals were utilized. As noted earlier, since this phase of the study, these organizations have been replaced by Primary Health Networks.

An *Invitation to Participate Letter* was sent to each potentially eligible GP site, which outlined the study and requested participation. The letter was addressed to the lead physician(s), owner(s), or manager at each address and was signed by the project Principal Investigator (PI) and two CIs (both general practitioners). Letters of invitation to the community health centers also included a letter of support from Monash Health, who governs the centers. A PULSAR team member made follow-up telephone calls four-to-seven days after letters were posted to explore interest and, if appropriate, request a meeting with senior site staff to explain the study further. If a GP decided to participate, she or he was required to sign the GP Participant Information and Consent Form.

Simultaneously, the study was advertised and GP participation sought using various newsletters and websites.

#### Adaptations to GP recruitment and retention

In addition to allowing more than one GP per cluster site to be recruited and expanding the geographical area of participating practices, as mentioned above, increased efforts were made to engage the Medicare Locals in promoting the study. This led to a sufficient number of GPs (*n =* 30) being recruited.

#### Patient recruitment

Participating GPs are responsible for organizing patient recruitment at their site. Recruitment is coordinated by the participating GP independently of the researchers, to maintain the privacy of patient participants and minimize the research-related administrative burden placed on practice site staff. Assistance is offered by the PULSAR researchers in training relevant practice staff in identifying potentially eligible patients.

GPs were requested initially to identify around 50 eligible patients from their practice site. Each cluster site was originally provided with a base remuneration of $200 for committing resources to help offset administration costs involved in recruitment. An additional $25 is received by the site for the successful recruitment of each eligible patient, and an additional $25 is sent to patients who return the survey in the provided return envelopes to the researchers.

Strategies to assist participating GPs are informed by the known impediments to recruitment in general practice [40–42]. The primary mode of patient recruitment is through survey packs that are sent in mail outs or handed out by each practice site. The packs contain an invitation to participate letter, a participant information and consent form, the study instruments, and two reply-paid envelopes. These envelopes are addressed to the PULSAR team rather than to patients’ clinics in order to reduce possible breaches of confidentiality; consenting participants return their data directly to the research team, who then securely enter (using password-protected files and computers) and store (using lockable filing cabinets for hard copies) all information. GPs and/or practice managers are required to oversee the mailing or handing out of survey packs.

Recruitment strategies were designed from the start to be flexibly employed according to the needs of each practice. A number of secondary strategies were also developed to promote patient response, including offering on-site availability of researchers, advertising via practice sites, and GPs handing out information flyers to eligible patients.

Practice billing and clinical software is used to identify those patients treated by the participating GP in the previous three months who have had a mental health plan drawn up or a review of a mental health plan conducted and/or have been prescribed any class of antidepressant medication on a continuing basis (for at least a month) as treatment for a mental illness. Billing and clinical software is also used to identify patients of the participating GP who have been prescribed antipsychotic medication in the previous six months or have had a diagnosis of psychosis. The participating GP is required to screen all lists of potential participants, with reference to their clinical notes if necessary, to confirm clinical status and eligibility for the study. It is emphasized that this step is important in the identification of patients with a diagnosis of psychosis as anti-psychotic medications are also prescribed for people without such a diagnosis.

#### Challenges with patient recruitment and retention

Initial patient recruitment by the cluster sites generated mixed results that varied from one cluster site completing baseline recruitment within one week to others not reaching their target after six months. Some clusters reported difficulties in identifying potentially eligible patients and a reluctance to mail to patients in case doing so jeopardized the practice/patient relationship. Other reasons for some cluster sites not distributing the survey to patients were: confusion about patient eligibility; believing the study was over or mistakenly thinking that the cluster had withdrawn from it; a lack of interest from patients in completing the surveys; and the required increase in administrative burden. Reasons given for being unable to identify eligible patients included resource pressures or having high numbers of patients that were either under the age of 18 or from culturally and linguistically diverse (CALD) backgrounds with low English proficiency.

#### Adaptations to patient recruitment

In addition to site visits, other strategies to assist clusters include an increase in baseline remuneration to $500, identification by the Project Manager of key personnel for liaison, and reductions of the administrative burden by the provision of relevant, clinic-tailored advice. A sub-study has been established to examine aspects of recovery-oriented practice in GP clinics with high CALD patient populations.

### Procedures to minimize bias

The study GPs could not be blinded to the intervention allocation as the intervention involved GP training. Therefore, the following procedures to minimize other sources of bias have been adopted:

1. Wherever possible, the research assistants, administration staff, and data entry staff are blinded to the intervention allocation.
2. Patients enrolled: procedures undertaken to minimize contamination include that participating patients are not advised by the research team if their GPs has received the intervention training.
3. Control clusters: patients in the control clusters continue to receive treatment as usual. As the study has a stepped-wedge design, all sites will receive the intervention by Step 2. Stepped-wedge designs are often preferred for such community-based pragmatic trials as they can minimize contamination of control clusters as staff and clinicians in all sites know that the study intervention is eventually coming [32–34].
4. Recruitment: capacity to give informed consent is presumed for the majority of people and provisions are made to ensure those most in need are given the opportunity to participate. For example, the recruitment sampling method outlined above allows for maximum numbers of patients to be identified who then have a choice in deciding whether or not they would like to participate in research. This is done to assist in avoiding possible sampling bias and gives flexibility for patients to respond based on possible fluctuations in mental health. Ethics research indicates that people with serious mental illness are able to provide informed consent to participate in research, especially if particular efforts are made to recruit them [50]. Research also indicates that, for people with mental health issues, participating in research can lead to positive reactions such as a sense of enjoyment and empowerment, and is beneficial for improved service delivery [51, 52]. It is presumed that those who do not wish to participate, or those who are unable to understand the study goals and procedures, will not return the consent forms and completed measures.
5. Allocation: all randomization was carried out by the statistician in the research team following the appropriate procedures set out earlier.
6. In analysis: wherever possible, the data collectors and data entry team are blinded to allocation status and do not have access to information about the allocation of clusters. Study data are entered into a study database that does not contain information about intervention status.

## Statistical analyses

The recovery-oriented practice training intervention for primary care will be evaluated at the patient level by examining the surveys of adult patients who consult the participating GPs for mental health issues. The main evaluation plan is to examine the cross-sectional surveys returned by patients during three data collection periods: baseline, Step 1, and Step 2 (see Fig. 1).

The planned data collection schedule depends on the training intervention being delivered at each cluster in the scheduled month (see Fig. 1). As training delays in some clusters are expected, the following two main analysis approaches are planned to compare the post-intervention and control (pre-intervention) periods:

1. Study planned dataset, which will use data collected from patients during the planned six-month periods centered on the midpoints of February 2015, August 2015 and November 2015 (see Fig. 1). Other data collected outside of these timespans are considered a protocol violation and are excluded from this dataset.
2. Per-protocol all data dataset, which will use all available data time-stamped from date of intervention received at the cluster, as outlined in Table 3.

### Main analysis plan

Descriptive statistics will be used to summarize the characteristics of the patient-level variables and the GP clinics (clusters), see Table 2. Cluster-level variables are those used in the stratified randomization, which are four types of organizational variations, plus the intervention status of the cluster and the time since (or before) the intervention. The intraclass correlation coefficient (ICC) will be calculated and reported.

**Table 2:** Individual and cluster-level variables available for multivariable analysis

Variable	Description
*Individual level*	
Demographics	
Sex	Sex of patient.
Age	Age of patient at survey completion date.
Country of birth	Country of birth of patient.
Ethnicity	Ethnic or cultural group that the patient identifies with.
Main language	Main language spoken at home.
Marital Status	Marital status of patient.
Living situation	Current living situation of patient.
Education	Education level of the patient.
Health Economics	
Mental health medications	Medications for mental health taken regularly by the patient.
Employment	Current working status of the patient.
Income	Usual weekly income of patient, after tax, from all sources of employment.
Days out of role	Number of days in the past month that the patient was totally or partly unable to carry out normal activities because of mental health problems.
Days absent from work	Number of days in the past month that the patient was absent from work due to illness or disability, and due to mental health problems.
Hospitalizations	Number of hospital admissions for physical problems and for mental health problems, including number of nights in total and reasons for most recent admissions.
Consultations with health professionals	Number and length of consultations with health professionals for physical health and mental health problems.
GUPI	The General-Practice Users’ Perceived-need Inventory is a one-page instrument developed to assess participants’ estimation of their needs for mental health care and the meeting of those needs.
*Cluster level*	
Cluster group	Allocated to receive the intervention at either Step 1 or Step 2.
Intervention status (0/1)	A lag time of six months is anticipated until intervention effects are possible. The intervention status variable indicates that this lag time has passed.
Dosage (%)	Intervention dosage.
Time since intervention	All patient surveys are time-stamped in relation to the time the intervention was received at the cluster. Time value of “0” is given for the plus/minus three months from date of training; “1” for four-to-six months post training; “2” for seven-to-nine months post training, etc. Time value of “-1” for four-to-six months before training; “-2” for seven-to-nine months before training, etc.
Time	Study month that survey was completed: “0” = December 2014, “1” = January 2015, etc.
*Cluster level stratification variables*	
Clinic type	Privately owned general practice vs community health center.
GP clinic size	GP equivalent full time (EFT) size greater than 5 (yes/no).
Clinic location	Local government area location: Dandenong, Casey, Cardinia, other.
Specialist focus clinic	A specialist focus clinic (yes/no) was determined by more than 10% of the practice patients belonging to any of the following groups: a. people with HIV/AIDS b. people with enduring and serious mental illness (e.g., schizophrenia, bipolar disorder) c. people with permanent physical disabilities d. people with addictions e. homeless people f. transient/seasonal populations g. people living in poverty h. Aboriginal peoples i. recent immigrants (six months or less) j. people from cultural minorities k. people with sports injuries l. other

The primary analysis will examine the patient-level QPR scores (continuous data) at baseline, nine months (Step 1), and 18 months (Step 2), using a linear mixed-effects model. The model will include intervention status and time as fixed effects and clusters and patients as random effects. Both univariate and multivariable models will be developed based on baseline patient and cluster-level variables considered statistically significant (*p <* 0.10) or clinically important (e.g., age, sex), see Table 2, and included in the model as fixed. This will include the intervention dosage variable described earlier. Model fit will be examined by comparing AIC values.

Secondary analyses will examine the patient-level data of the INSPIRE, WEMWBS, and K10. Similar to the primary analysis, we will use linear mixed-effects models to compare the intervention and control periods (pre-intervention) for continuous outcomes and generalized linear mixed-effects models for binary outcomes.

Estimated intervention effects will be reported as the mean outcome difference for continuous outcomes and Odds Ratio for binary outcomes between intervention and control periods, assuming a constant treatment effect over time. This can be described as a meta-analysis approach as (in the case of continuous data) the mean change in each cluster will be standardized by using the variance of the outcome measure within that cluster. The estimated intervention effects will be reported with 95% Confidence Intervals and *p* values. Analysis will be conducted using Stata V.14, StataCorp. Stata Statistical Software: Release 14. College Station, TX: StataCorp LP, 2015.

### Sensitivity analyses

A missing data analysis will investigate any patterns of missingness. For each primary and secondary outcome component with missing data, multiple imputation using multivariate regression with factors of age, gender, time, and intervention status will produce 100 estimates. Sensitivity analyses will be performed using this multiple imputation to account for missing data and then re-running the analyses.

#### Economic evaluation

Overall, costs associated with each participant will follow well established health economic principles [53], and cover direct medical costs of illness, plus the labor market effects of illness. Direct medical costs are to be calculated for prescription medications and hospital and health service contacts. Labor market productivity losses will be imputed using the human capital approach by multiplying reported days off work due to mental illness with an individual’s estimated salary using instrumentation devised by this team for a previous health economic evaluation [54]. Only using days off work due to illness to capture labor market costs captures an important aspect of the cost of illness; however, it is noted that the estimates obtained will be conservative and the true cost will be higher than what we obtain because of other effects of illness such as higher rates of non-participation in employment, or underemployment.

### Data collection and management

Table 3 outlines the data collected at each time point for participating GPs and patients. Patient participant data is being collected by the returned paper surveys or online surveys at three time points: baseline (months 4–6), Step 1 (months 16–18) and Step 2 (months 28–30), as displayed in Fig. 1. Both modes of survey completion are provided to offer flexible participation options. The estimated completion time for each round of surveys is approximately 45 min.

**Table 3:** Schedule of enrolment, interventions, and assessments

	Time points			
Project events		T0	T1	T2
Intervention adaptation	X			
GP enrolment				
Eligibility screen	X			
Informed consent	X			
Randomization	X			
GP training intervention				
Year 1 clusters		X		
Year 2 clusters			X	
GP PALS				
Year 1 clusters		X	X	X
Year 2 clusters			X	X
Patient recruitment				
Eligibility screen by GP		X	X	X
Survey packs provided to eligible patients via GPs		X	X	X
Informed consent		X	X	X
Patient quantitative assessment				
Demographics		X	X	X
QPR		X	X	X
WEMWBS		X	X	X
INSPIRE		X	X	X
K10		X	X	X
Health economic items		X	X	X
GUPI		X	X	X
GP qualitative assessment				
Informed consent			X	X
Individual interview			X	
Individual interview/focus group				X
Patient qualitative assessment				
Informed consent			X	X
Individual interview			X	
Individual interview/focus group				X

The online survey option collects participant-entered data directly from Qualtrics online survey software. Qualtrics securely collects participant responses within a secure back-end spreadsheet that is only accessible by the researchers via password protected files.

If patient participants consent to allowing the researchers to access their routinely collected medical records, additional information will be extracted from these files with the assistance of the participating GP/s in each cluster site, including information about diagnosis and mental health status and medication details relating to mental health issues.

All study data is stored in re-identifiable format, from which identifiers have been removed and replaced by a code. Re-identification is necessary in order to link survey data with routinely collected data for patient participants who consent to the release of this information. Any personal information such as participant names remain confidential and all information is stored in password protected files and folders on password protected computers located at the core PULSAR administration site. These can only be accessed by the research staff. The study data will be stored for a minimum of 7 years, after this time it may be confidentially destroyed. It may be possible that future research such as a meta-analysis will use the participant data in non-identifiable format.

### Qualitative methods

The embedded qualitative study was designed to investigate the contextual enablers and challenges to implementing recovery-oriented practice in participating primary care settings through two stages of qualitative data collection. First, an interview schedule based on the Promoting Action on Research Implementation in Health Services (PARIHS) framework [55, 56] was used with a small number of GPs to identify potential barriers or challenges to delivering recovery-oriented practice training for GPs. This approach enabled the intervention sites’ readiness for recovery-oriented practice to be gauged, and the optimization of the intervention package and its implementation.

Second, semi-structured interview schedules, informed by literature on the implementation of recovery-oriented practice and consumer and service provider expertise within the PULSAR Qualitative Research Steering Group, were developed to investigate GP and patient views and experiences of recovery-oriented practice within primary care settings. These interviews are conducted face-to-face or by telephone at three and nine months following the GP training. Interviews with GPs explore their understanding of recovery-oriented practice and experiences and challenges encountered in implementing a recovery-oriented framework at practice level. Subsequently, participating GPs are invited to reflect on the de-identified interview themes, and on facilitators and barriers to implementing recovery-oriented practice in primary care settings, in an interview or focus group discussion. Similarly, face-to-face or telephone interviews conducted with patient participants focus on their views and experiences of recovery-oriented practices as used by GPs, with a subsequent opportunity to reflect on the de-identified interview themes and on supports for their recovery within and beyond services.

Sample size is determined sequentially by qualitative sampling processes to ensure diverse perspectives are sought, and to maximize the richness of data obtained, for which we anticipate at least 10 GP participants and 10 patient participants will be interviewed.

All qualitative data are audio-recorded (subject to participant consent) or documented in handwritten notes, then transcribed for coding and thematic analyses, so as to identify thematic similarities and differences within and across participant groups. All transcribed data are de-identified and along with all other PULSAR data are stored in password-protected files within the restricted access electronic files of the PULSAR site.

### Process evaluation

The Project Team developed a fixed schedule of content for the training sessions for GPs and actively referred to this schedule during delivery. Following the guidelines provided by Moore, Audrey, Barker, Bond, Bonell, Hardeman et al. the process evaluation will focus on collecting data in the following four areas:

1. Description of the intervention and project assumptions (Module 1: Adaptation)
2. The implementation context (Module 2: Implementation)
3. Mechanisms of impact (Module 3: Research)
4. The outcomes of the study (Module 4: Dissemination).

This evaluation will yield data on the process of implementing a complex intervention design associated with the stepped-wedge approach such as sampling responses, effect sizes, and organizational and contextual factors that impact the study processes and outcomes. Qualitative and quantitative analysis provide evidence of the influence of contextual and implementation factors on outcomes. Together, the data will be integrated to identify barriers and enablers for achieving expected outcomes from the intervention and contribute explanatory detail with respect to main findings.

#### Monitoring

A project implementation group (which functions as a *data monitoring committee*) meets weekly, monitoring progress towards targets set by the module task groups, reporting and escalating issues to those task groups as necessary. These meetings are attended by the principle investigator, project manager and other core study staff. In these weekly meetings, protocols prepared or amended by the module task groups are operationalized to provide guidance to the study research team. Feedback is also collated on intervention implementation, maintenance, and monitoring of the overall conduct of the trial; this information is readily relayed to the relevant module task groups and principle investigator, thereby enabling timely assessment and intervention if necessary.

#### Leadership structure

The management and advisory structure of the PULSAR project adopts a module based approach. The trial is overseen by a project steering group (Chair: Professor Graham Meadows) and four modules guide and support the development, implementation and evaluation of the project. The module task groups and chairs are: Adaptation module (Chair: Christine Thornton & Graham Meadows); Implementation module (Chair: Penny Weller); Research module (Chair: Lisa Brophy); and Dissemination module (Chair: Vrinda Edan). The Lived Experience Advisory Panel (LEAP) is a consumer and family/carer advisory group that guided the development of the REFOCUS-PULSAR materials and training intervention and provides ongoing recommendations on the implementation of the trial.

#### Specification of safety parameters

No plans were made for a premature stopping of the trial. Participant (GP and patients) safety was classified as low risk, apart from any breaches to patient confidentiality, which were classified as moderate risk.

#### Safety oversight

Detailed project protocols have been developed addressing the management of participant distress, suicidal ideation or intent, threat to harm others, and disclosure of previously undisclosed criminal acts.

## Discussion

A stepped-wedge cluster randomized controlled trial in the general practice and community health center primary care settings is an innovative and novel approach to test a training intervention for general practitioners (GPs) in recovery-oriented practice. This intervention will enable GPs, and their staff, who provide primary care to people with mental illness in this study to be introduced to recovery-oriented practice and to recruit the people who consult them to be actively engaged in evaluating the effectiveness of the intervention through a mixed methods study design. This research design has both advantages and limitations [57], and the challenges encountered are providing important insights into methods to maximize the recruitment and retention of GPs in a large scale and complex study, as well as how to engage the patients of GPs who are presenting with mental ill health. If findings are positive from the work described, the intervention has the potential to extend recovery-oriented practice to GPs and thus reduce the current barriers to GPs and their patients gaining access to this system-wide transformation.

### Dissemination policy

#### Overview

A multi-level approach to knowledge transfer towards influencing practice will be used. The plan will include: publication of a training manual and information leaflets; submission for publication in peer-reviewed literature of findings from each component of the project; presentations at international and national scientific and practice-focused conferences; web-site development for making project materials readily accessible to other interested parties; local dissemination through our partner organizations in Victoria’s mental health treatment and support sectors; and direct presentations to policy makers to ensure the findings are well understood and appreciated where key decisions are being taken.

#### Rights

In relation to copyright issues in dissemination of findings, PI Meadows and CI Slade have agreed to highly accessible publication to maximize dissemination. Specifically, there is no plan to commercialize outputs of this work and so put barriers in the way of use by others. It has been the practice of the multiple research teams involved in the PULSAR proposal actively to seek to make materials widely available without cost, and to place barriers in the way of others commercializing such work. For example, the London REFOCUS team have disseminated the REFOCUS intervention in free-to-access booklets and through open access journal articles. The dissemination plan will make the findings widely and readily available along with source training materials.

## Abbreviations

ANZCTR: Australian New Zealand Clinical Trials Registry
CI: Chief Investigator
cRCT: Cluster randomized control trial
GPMHTP-R: General practitioner mental health treatment plan-recovery
GUPI: General-practiceusers' perceived-need inventory
HREC: Human research ethics committee
ICC: Intraclass correlation coefficient
LEAP: Lived experience advisory panel
MHST: Mental health skills training
NHMRC: National health and medical research council
PARIHS: Promoting action on research implementation in health services
PI: Principal investigator
PULSAR: Principles unite local services assisting recovery
QPR: Questionnaire about the process of recovery
SWCRCT: Stepped-wedge cluster randomized controlled trial
WEMWBS: Warwick-edinburgh mental well-being scale
